# Comparative Vascular Effects of Sirolimus and Everolimus on Isolated Human Saphenous Veins

**DOI:** 10.3390/life15040553

**Published:** 2025-03-28

**Authors:** Deniz Kaleli Durman, Erkan Civelek, Fatoş İlkay Alp Yildirim, Önder Teskin, Birsel Sönmez Uydeş Doğan

**Affiliations:** 1 Department of Pharmacology, Faculty of Pharmacy, Istanbul University, 34116 Istanbul, Türkiye; 2 Department of Cardiovascular Surgery, Faculty of Medicine, Biruni University, 34015 Istanbul, Türkiye

**Keywords:** drug-eluting stents, sirolimus, everolimus, human saphenous vein, vascular tone

## Abstract

Drug-eluting stents, which release antiproliferative agents such as sirolimus and everolimus, were developed to reduce the risk of restenosis associated with bare-metal stents. However, despite their proven clinical efficacy, concerns remain regarding in-stent restenosis due to delayed endothelial healing and the risk of late thrombotic events. In this study, we aimed to determine the vascular effects of sirolimus and everolimus on isolated human saphenous vein (SV) samples obtained from patients undergoing coronary artery bypass surgery. SV rings were subjected to sirolimus and everolimus in acute and pretreatment conditions in vitro. Increasing concentrations of sirolimus (10^−8^–10^−5^ M), everolimus (10^−8^–10^−5^ M), and their vehicle were administered to SV rings precontracted with phenylephrine (Phe,10^−6^–5 × 10^−6^ M) to evaluate their direct vascular effects. Additionally, SV rings were incubated (16 h) either with sirolimus (10^−5^ M), everolimus (10^−6^ M), or the vehicle. Thereafter, the contractile responses to Phe (10^−8^–10^−4^ M), and the endothelium-dependent and endothelium-independent relaxant responses to acetylcholine (ACh, 10^−8^–10^−4^ M) and sodium nitroprusside (SNP,10^−8^–10^−4^ M) were determined, respectively. Our findings demonstrated that sirolimus and everolimus did not exert direct relaxant and modulatory effects on vascular function in isolated human SVs. Hence, the preservation of contractile and relaxant responses with sirolimus and everolimus may have clinical implications in the context of DES implantation.

## 1. Introduction

Percutaneous coronary interventions (PCI) with stent implantation have provided an efficacious treatment of coronary artery disease by improving patient outcomes [1,2]. Nevertheless, in-stent restenosis is a significant clinical issue, primarily driven by the proliferation of vascular smooth muscle cells and inflammatory responses leading to neointimal hyperplasia [3,4]. Drug-eluting stents (DESs), which release antiproliferative agents such as sirolimus and everolimus, were developed to reduce the risk of restenosis associated with bare-metal stents (BMSs). However, despite their proven clinical efficacy, concerns still remain regarding in-stent restenosis due to delayed endothelial healing and the risk of late thrombotic events. Given that DESs consist of components, namely the stent platform, antiproliferative drug, and polymer, any of these elements may have the potential to induce in-stent restenosis. This highlights the need for further research into the direct vascular effects of the eluted drugs, particularly in the absence of the platform and polymer components of DESs [5,6,7,8,9,10,11].

Numerous animal studies have investigated the vascular effects of antiproliferative agents used in DESs, particularly sirolimus, in both in vivo and in vitro settings. These studies have employed various study protocols, drug dosages, and treatment durations, and displayed conflicting results in terms of the effects of these agents on endothelial function and vascular reactivity to vasodilator and vasoconstrictor agents [12,13,14,15,16]. Notably, only a limited number of studies so far have evaluated the vascular effects of sirolimus on human arteries, namely isolated human radial arteries and internal thoracic arteries (ITAs), which demonstrated its vasorelaxant effect and modulatory influence on endothelial function, respectively [17,18]. While arterial grafts, namely the ITA and radial artery, are generally preferred in coronary artery bypass graft (CABG) surgery due to their better long-term patency rates, the use of saphenous veins (SVs) remains widespread, particularly in cases where multiple grafts are required, or when arterial grafts are unavailable or unsuitable. However, SV grafts are more prone to complications, including vasospasm, thrombosis, and early graft failure due to restenosis. Studies indicate that approximately 50% of SV grafts fail within 10 years post-CABG, primarily due to restenosis, intimal hyperplasia, and graft occlusion [19,20,21]. These challenges highlight the need for further investigation into the effects of DESs in SVs, which may offer a strategy to mitigate these risks and improve the long-term success of SV grafts in coronary revascularization. Indeed, the use of DESs in SV graft lesions has been proposed to reduce the incidence of restenosis and improve long-term clinical outcomes in patients with atherosclerotic cardiovascular disease [22,23]. While prior in vitro studies have examined the effects of sirolimus in human arterial grafts, the direct vascular effects of sirolimus and everolimus on isolated human SVs remain unexplored. Therefore, in this study, we specifically focused on SVs to better understand how these drugs influence vascular tone and function in a graft material prone to unique complications.

The current study aims to investigate the vascular effects of sirolimus and everolimus on human isolated SV rings under both acute and pretreatment conditions. For this purpose, the acute effects of these agents were evaluated in phenylephrine-precontracted SV rings, whereas their influence on endothelial function and vascular reactivity was assessed in SV rings pretreated with either sirolimus or everolimus.

## 2. Material and Methods

### 2.1. Harvesting and Preparation of Saphenous Veins

SV samples were harvested from patients undergoing coronary artery bypass operations. The use of discarded human SV samples was approved by the Institutional Review Board of Haseki Research and Training Hospital, Istanbul, Türkiye (2012/373). Informed consent was obtained from all patients who participated voluntarily in the study. The study was conducted in accordance with the principles outlined in the Declaration of Helsinki.

Caution was exercised during harvesting of a vessel in order not to stretch and touch the endothelial surface. After the harvesting procedure, human SV samples were placed in cold (4 °C) Krebs-Ringer bicarbonate solution and transferred to the laboratory immediately. The composition of the Krebs-Ringer solution was as follows (in mM): NaCl 118.5, KCl 4.8, KH_2_PO_4_ 1.2, NaHCO_3_ 25, MgSO_4_.7H_2_O 1.2, CaCl_2_ 1.9, glucose 10.1, and disodium EDTA 0.026. Adherent connective and adipose tissues were removed carefully and specimens were cut into 3–4 mm length rings. Three to four rings were obtained from each vein specimen. Isolated SV rings were placed into an organ bath system containing Krebs-Ringer bicarbonate solution at 37 °C, aerated with 95% O_2_ and 5% CO_2_, and suspended between two stainless steel L-shaped hooks. One hook was fixed at the bottom of the organ bath while the other was connected to a force displacement transducer (Grass Model FT03; Grass Telefactor, West Warwick, RI, USA). Rings were equilibrated for 2 h at 2 g resting tension. Thereafter, 40 mM potassium chloride (KCl) was administered to determine the viability of vein rings, and preparations that produced a tension of less than 2 g were discarded. Two consecutive KCl (40 mM) responses were obtained for each preparation to standardize the reactivity of the vessel rings.

### 2.2. Experimental Protocol

**Figure 1:**
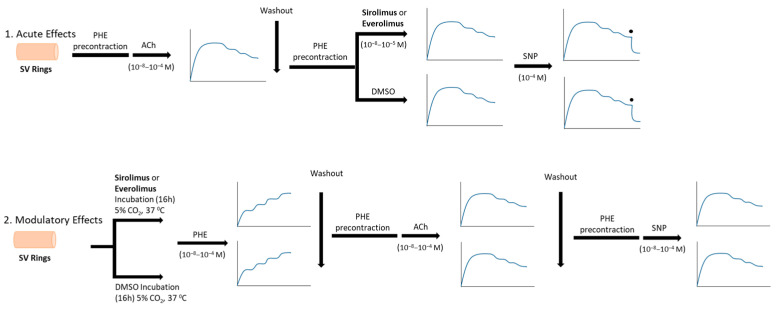
Schematic overview of the experimental protocols designed to evaluate the acute and modulatory vascular effects of sirolimus and everolimus in isolated human SV rings.

This study was designed to evaluate the vascular effects of sirolimus and everolimus in isolated human SV rings under both acute and pretreatment conditions by using two different experimental protocols. In the first set of experiments, the acute vascular effects of these agents were studied in SV rings precontracted with submaximal concentrations of phenylephrine (Phe, 10^−6^–5 × 10^−6^ M) as appropriate. In the second set of experiments, their modulatory vascular effects on endothelial function and vascular reactivity were assessed in SV rings following a 16 h incubation at 37 °C under conditions of 95% O_2_ and 5% CO_2_. This dual approach enabled the investigation of both acute direct vascular effects and potential modulatory vascular effects induced by sirolimus and everolimus in human SV rings. The experimental protocol is summarized below (Figure 1).

#### 2.2.1. Experimental Protocol for Acute Effects of Sirolimus and Everolimus

After the standardization of isolated SV rings with KCl (40 mM), the presence of functional endothelium was confirmed by the relaxation response to the endothelium-dependent vasodilator acetylcholine (ACh, 10^−8^–10^−4^ M) in rings precontracted with Phe (10^−6^–5 × 10^−6^ M). Thereafter, increasing concentrations of either sirolimus (10^−8^–10^−5^ M) or everolimus (10^−8^–10^−5^ M) were applied in a cumulative manner on Phe precontracted SV rings. In parallel rings of each specimen, the effect of the vehicle for sirolimus and everolimus, namely DMSO (<0.1%), was also determined. Additionally, time-match control experiments were also performed in preliminary studies to elucidate whether the precontractions elicited by Phe were stable during the experimental period. At the end of the experiments, sodium nitroprusside (SNP, 10^−4^ M) was administered to each vessel ring to determine the maximum smooth muscle relaxation capacity.

#### 2.2.2. Experimental Protocol for Modulatory Effects of Sirolimus and Everolimus

Isolated SV rings were weighed and placed into 1.5 mL Eppendorf tubes containing 70 mg tissue/1 mL RPMI 1640 medium supplemented with Penicillin-Streptomycin-Amphotericin (PSA) solution (1% *v*/*v*). The SV rings were then incubated overnight (16 h, 37 °C) under 95% O_2_ and 5% CO_2_ conditions in the presence of either sirolimus (10^−5^ M), everolimus (10^−6^ M), or DMSO (vehicle). At the end of the incubation period, SV rings were transferred to an isolated organ bath system to evaluate the modulatory effects of sirolimus and everolimus on vascular endothelial function and contractile reactivity. Following the standardization of SV rings with KCl (40 mM), ACh (10^−8^–10^−4^ M) and SNP (10^−8^–10^−4^ M) were cumulatively administered to Phe–precontracted (10^−6^–5 × 10^−6^ M) rings to evaluate the effects of sirolimus and everolimus on endothelium-dependent and endothelium-independent relaxations, respectively. Additionally, increasing concentrations of Phe (10^−8^–10^−4^ M) were cumulatively applied to SV rings to determine the influence of sirolimus and everolimus on vascular contractile reactivity.

### 2.3. Statistical Analysis

All data are presented as mean ± standard error of the mean (SEM). In all sets of experiments, “n” represents the number of patients from whom the SVs were obtained. Relaxation responses to sirolimus, everolimus, SNP, and ACh are expressed as % decreases in Phe–induced precontraction, while contractile responses to Phe are presented as the % of 40 mM KCl–induced contractions. E_max_ denotes the maximal relaxation or contraction responses to these agents. The sensitivities of SV rings to relaxant agents (ACh and SNP), everolimus, and sirolimus, as well as the contractile agent (Phe) were determined as the effective concentration that elicited 50% (EC_50_) of the maximal response, calculated separately for each concentration–response curve by probit regression analysis, and expressed as –log M (pEC_50_). Statistical analysis was performed by two-way analysis of variance (ANOVA), and a *p*-value of less than 0.05 was considered statistically significant. GraphPad Prism software (version 9.4.0, Windows, Boston, MA, USA) was used for all statistical analyses. 

### 2.4. Chemicals

PSA solution and RPMI 1640 medium were purchased from PAN-Biotech (Aidenbach, Germany). All other agents were obtained from Sigma-Aldrich Co. (St. Louis, MO, USA). Stock solutions of sirolimus and everolimus were prepared in DMSO and further diluted in Krebs-Ringer solution. The final concentration of DMSO in the organ bath or incubation medium was <0.1%. A stock solution of ACh was prepared in 0.001 N HCl whereas SNP, Phe, and KCl were prepared in distilled water. All dilutions were freshly prepared in Krebs-Ringer solution on the day of each experiment.

## 3. Results

### 3.1. Patient Characteristics

The clinical characteristics of patients undergoing coronary artery bypass surgery and their medication regimens are presented in Table 1. The majority of the patients (67% male and 33% female) exhibited at least one cardiovascular risk factor, including hypercholesterolemia, hypertension, and diabetes mellitus. Regarding preoperative medication use, β-blockers and diuretics were the most commonly prescribed drug classes, followed by statins (HMG-CoA reductase inhibitors) and ACE inhibitors. Additionally, during the operation, 96% of patients received nitrovasodilators, while 4% were administered calcium channel blockers. Clinical characteristics of the patients and their drug therapies are given in Table 1.

### 3.2. Acute Effects of Sirolimus and Everolimus

**Figure 2:**
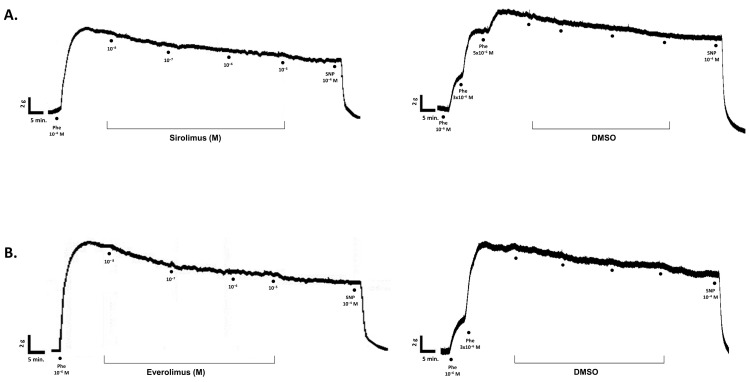
Representative original tracings illustrating the effects of sirolimus (**A**), everolimus (**B**), and their corresponding vehicle controls (i.e., DMSO) on phenylephrine (10^−6^–5 × 10^−6^ M)-precontracted human SV rings.

**Figure 3:**
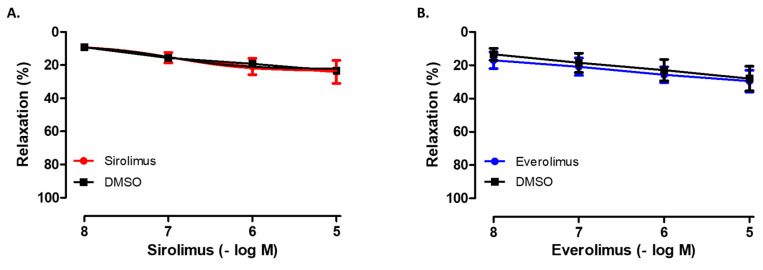
Concentration-dependent effects of sirolimus (10^−8^–10^−5^ M) (**A**) and everolimus (10^−8^–10^−5^ M) (**B**) on phenylephrine (10^−6^–5 × 10^−6^ M)-precontracted SV rings. The influence of the vehicle (i.e., DMSO) is also shown for each agent. The relaxation responses are given as the % of phenylephrine-induced precontraction. Values are given as mean ± SEM (n = 5). Two-way ANOVA is performed for statistical analysis.

As shown in Figure 2 and Figure 3, increasing concentrations of both sirolimus (10^−8^–10^−5^ M) and everolimus (10^−8^–10^−5^ M) produced weak relaxant responses in isolated SV rings, which were comparable to those induced by the vehicle, DMSO (*p* > 0.05) (Table 2). Moreover, time-matched control studies demonstrated that the precontractile tone induced by Phe (10^−6^–5 × 10^−6^ M) remained stable throughout the experimental period.

### 3.3. Modulatory Effects of Sirolimus and Everolimus

**Figure 4:**
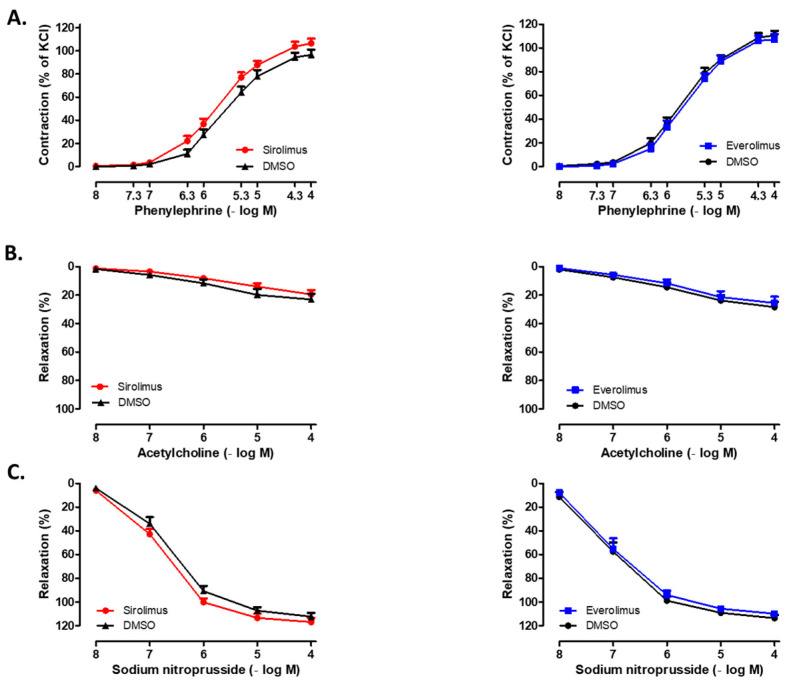
Concentration-dependent effects of (**A**) phenylephrine (10^−8^–10^−4^ M), (**B**) acetylcholine (10^−8^–10^−4^ M), and (**C**) sodium nitroprusside (10^−8^–10^−4^ M) on isolated human SV rings incubated overnight (16 h) with either sirolimus (10^−5^ M) or everolimus (10^−6^ M). The influence of vehicle DMSO incubation is also shown for each agent. Responses to phenylephrine are expressed as the % of 40 mM KCl–induced contractions, whereas relaxation responses to sodium nitroprusside and acetylcholine are given as the % of phenylephrine-induced precontractions. Values are means ± SEM (n = 9–14). Two-way ANOVA is performed for statistical analysis.

Overnight incubation (16 h) of isolated human SV rings with sirolimus (10^−5^ M) or everolimus (10^−6^ M) did not significantly alter contractile responses to Phe (10^−8^–10^−4^ M). In addition, endothelium-dependent relaxations to ACh (10^−8^–10^−4^ M) and endothelium-independent relaxations to SNP (10^−8^–10^−4^ M) in Phe–precontracted rings were not modified in the presence of either sirolimus or everolimus. Moreover, incubation of SV rings with DMSO also did not modify contractile and relaxant responses (*p* > 0.05) (Figure 4, Table 3).

## 4. Discussion

The present study originally demonstrated that increasing concentrations of sirolimus and everolimus did not have a direct vasorelaxant effect in isolated human SV rings. In addition, in vitro pretreatment (16 h) of SV rings with either sirolimus or everolimus did not significantly alter contractile reactivity to Phe or modify endothelium-dependent and endothelium-independent vasorelaxation responses to ACh and SNP, respectively. These findings suggest that sirolimus and everolimus do not have a direct influence on vascular tone in isolated human SVs.

A limited number of studies so far have investigated the effects of sirolimus on vascular tone in vitro, but yet no data are available for everolimus. In relation, acute vasorelaxant effects of sirolimus have been reported in rat aortic rings with endothelium as well as in isolated human radial arterial rings without endothelium [12,17]. Notably, sirolimus-induced relaxation in an isolated rat aorta was attributed to the modulation of its vasoactive vehicles, polyethylene glycol (PEG) and fat emulsion (intralipid) [12]. However, in the latter study on isolated human radial arteries, direct influence of the vehicle for sirolimus (DMSO) was not indicated [17]. In the present study, the vascular effects of sirolimus and everolimus were determined in parallel to their vehicle, DMSO. Our findings clearly demonstrated that both sirolimus and everolimus do not have a direct vasorelaxant effect on Phe–precontracted isolated human SV rings. To the best of our knowledge, this is the first study comparing the direct vascular effects of sirolimus and everolimus in human vessels, in particular, isolated SV rings.

Moreover, a few incubation studies have examined the vascular modulatory effects of sirolimus on endothelium-dependent and endothelium-independent relaxant responses as well as on spasmogen-induced contractile responses. No change [14] or a decrease [18,24,25] in endothelium-dependent relaxation to ACh has been reported in various arteries following pretreatment with sirolimus at different in vitro incubation periods. Similarly, in the current study, following an overnight (16 h) incubation with either sirolimus or everolimus, we determined that ACh–induced relaxation responses were unmodified, indicating that these agents do not influence endothelial function in an in vitro setting in isolated human SVs. Our findings with sirolimus are consistent with a previous in vitro study in rat aortic rings [14], whereas other studies in rat aortas [25], porcine coronary arteries [24], and isolated human IMAs [18] have reported an attenuated response to ACh following incubation with sirolimus. The impairment in endothelial function following incubation with sirolimus was attributed to a reduction in NO production [18,25], without any histological damage on the endothelium [18]. On the other hand, in vivo administration of sirolimus or everolimus have also shown conflicting results regarding endothelial-dependent vascular relaxation responses to ACh. In relation, either an increase [13,26], a decrease [16,25], or no change [14,15,16,27,28] in vascular endothelial function were reported. Notably, our study is the first to demonstrate the effects of both sirolimus and everolimus on the endothelial function of isolated human SVs. Differences in study protocol, vessel type, drug concentration/dosage, or incubation period/treatment duration could account for the differences observed between our findings and those of previous studies. Preserved endothelial function following sirolimus and everolimus pretreatment may have a favorable clinical impact on an SV graft, which is more prone to restenosis due to weak endothelial capacity compared to arterial grafts. However, these findings require further validation under in vivo conditions through clinical studies.

In addition, in the current study, following an overnight (16 h) incubation with either sirolimus or everolimus, we determined that the endothelium-independent relaxation responses to SNP were unmodified, suggesting that pretreatment with these agents does not modify the vascular relaxation capacities of isolated human SVs. These findings are consistent with previous experimental studies reporting that sirolimus [14,24,25] and tacrolimus [24] did not change SNP–induced relaxation responses in isolated rat aortas and porcine coronary arteries. Supportively, in vivo long-term treatment studies performed either with sirolimus or everolimus displayed no change in SNP–induced vascular relaxation responses in rat aortas [15,16,27]. Likewise, a clinical study in patients with a DES demonstrated that the vasodilator response to nitroglycerin was maintained around the vessel segments of sirolimus-eluting stents [29]. Therefore, our findings regarding the preservation of SNP–induced responses with either sirolimus or everolimus pretreatment may have clinical implications in the context of DES implantation.

Concerning vascular reactivity, contractions to increasing concentrations of Phe were not modified in either sirolimus- or everolimus-pretreated SV rings in line with previous in vitro studies, which used either similar or higher concentrations of sirolimus in isolated rat aortas [12,14] and human IMAs [18]. Supportively, despite a few studies reporting either enhanced [16] or reduced [15] contractile responses to noradrenaline (NA) in a rat aorta following sirolimus treatment (~ 2 weeks), the majority of other in vivo animal studies have shown no change in vascular reactivity to spasmogens, including NA and endothelin-1 (ET-1), following treatment (2–4 weeks) with sirolimus [14,16,27,28] and/or everolimus [16,30]. Based on the current in vitro findings in regard to maintained vascular reactivity to Phe in the presence of sirolimus and everolimus, it is reasonable to suggest that these agents do not appear to enhance susceptibility to vasospasm in human SVs. Notably, a previous clinical study documented exercise-induced vasoconstriction of coronary artery segments adjacent to a sirolimus-eluting stent, but these observations were suggested to depend on endothelial dysfunction as the underlying mechanism [29]. Clinical data on the use of DESs in patients with vein graft diseases are limited and give conflicting results in terms of the efficacity and safety of DESs. Comparative clinical studies with DESs and BMSs displayed superior patient outcomes in favor of DESs in the short-term, while similar patient outcomes associated with high rates of in-stent restenosis were reported for both DESs and BMSs in long-term follow-up [20,22,31,32,33]. Indeed, our findings are aligned with clinical studies demonstrating the short-term benefits of DES implantation in SVs without providing insight into the long-term effects of DESs.

Moreover, this study was conducted under in vitro conditions, which do not fully mimic the complex in vivo environment where shear stress, pulsatile blood flow, circulating immune cells, and systemic inflammatory mediators play a critical role in vascular responses. On the other hand, in vitro studies provide essential mechanistic insights by isolating the direct effects of these drugs without the confounding influences of systemic physiological factors. Thus, our in vitro findings provide valuable insights into the direct vascular effects of sirolimus and everolimus on isolated human SVs.

## 5. Conclusions

Although DESs effectively reduce restenosis compared to BMSs, concerns persist regarding delayed endothelial healing and in-stent restenosis. However, our findings demonstrate that sirolimus and everolimus do not exert direct relaxant or modulatory effects on vascular function in isolated human SVs. Contractile responses to Phe as well as the endothelium-dependent and endothelium-independent relaxant responses to ACh and SNP were preserved in SV rings following in vitro pretreatment with these agents. Given the crucial role of endothelial integrity in SV graft patency, our findings with sirolimus and everolimus on endothelial-dependent responses may provide supportive evidence for positive patient outcomes obtained in short-term clinical studies. Additionally, their lack of modulatory influence on spasmogen-induced contractions eliminates the risk of vasospasm in SVs, which further supports their vascular safety profile. These in vitro findings may provide a better understanding of the effects of sirolimus or everolimus in human SVs, though further clinical studies will be intriguing to confirm these experimental data in regard to their long-term vascular effects in patients with DES implantation.

## Figures and Tables

**Table 1:** Clinical characteristics of the patients undergoing a coronary artery bypass operation.

Parameters	n (%)
Age (year)	58.25 ± 1.93
Number of Patients	24
Sex	
- Male	16 (67%)
- Female	8 (33%)
**Diseases**	
Hypercholesterolemia	15 (63%)
Hypertension	19 (79%)
Diabetes Mellitus	12 (50%)
**Drug therapy**	
β-blockers	20 (83%)
ACE Inhibitors	1 (4%)
Diuretics	20 (83%)
Statins	3 (13%)
**Drug Therapy During Operation**	
Calcium Channel Blockers	1 (4%)
Nitrovasodilators	23 (96%)

n: the number of patients, ACE: angiotensin-converting enzyme.

**Table 2:** The maximal relaxation (E_max_) and pEC_50_ values of sirolimus (10^−8^–10^−5^ M), everolimus (10^−8^–10^−5^ M), and their vehicle (DMSO) on isolated human SV rings precontracted submaximally with Phe (10^−6^–5 × 10^−6^ M) as appropriate.

	E_max_ (%)	pEC_50_
Sirolimus	24.00 ± 6.71	6.97 ± 0.23
DMSO	24.36 ± 1.53	6.91 ± 0.45
Everolimus	30.91 ± 6.95	6.38 ± 0.25
DMSO	28.96 ± 7.12	6.41 ± 0.29

All results are expressed as mean ± S.E.M. E_max_ values are expressed as the percentage (%) of Phe–induced precontractions. EC_50_ values are expressed as—log EC_50_ (i.e., pEC_50_). For each group, n = 5.

**Table 3:** The influence of pretreatment (16 h) with sirolimus (10^−5^ M), everolimus (10^−6^ M), or their vehicle (DMSO) on Phe–induced contractions as well as endothelium-dependent and endothelium-independent relaxation responses to ACh and SNP, respectively, on isolated human SV rings.

	Phe		ACh		SNP	
	E_max_ (%)	pEC_50_	E_max_ (%)	pEC_50_	E_max_ (%)	pEC_50_
Sirolimus	108.1 ± 3.70	5.72 ± 0.07	20.1 ± 2.93	5.51 ± 0.18	116.8 ± 3.89	6.81 ± 0.07
DMSO	100 ±4.51	5.57 ± 0.07	23.29 ± 4.08	5.84 ± 0.17	112 ± 3.16	6.68 ± 0.1
Everolimus	113 ± 3.49	5.67 ± 0.06	29.79 ± 3.9	5.81 ± 0.23	112.6 ± 2.93	7.12 ± 0.15
DMSO	110.8 ± 4.41	5.63 ± 0.07	26.85 ± 4.61	5.78 ± 0.28	108.6 ± 2.64	7.2 ± 0.22

All results are expressed as mean ± S.E.M. E_max_ values represent the maximum responses (contraction or relaxation) to Phe, ACh, and SNP on isolated human SV rings incubated with sirolimus (10^−5^ M), everolimus (10^−6^ M), or their vehicle (DMSO). EC_50_ values are expressed as—log EC_50_ (i.e., pEC_50_); n = 9–14.

## Data Availability

The data presented in this study are available on request from the corresponding author.
